# Zugelassene Systemtherapien in der Dermatologie

**DOI:** 10.1007/s00105-021-04816-2

**Published:** 2021-04-21

**Authors:** Monika Kleinhans, Carolin Funke-Lorenz, Joachim Dissemond

**Affiliations:** Klinik und Poliklinik für Dermatologie, Venerologie und Allergologie, Universitätsklinikum Essen, Hufelandstr. 55, 45147 Essen, Deutschland

**Keywords:** Off-label-Use, Orphan Diseases, Compassionate Use Program, Early Access Program, Kostenerstattung, Off-label use, Orphan diseases, Compassionate Use Program, Early Access Program, Reimbursement

## Abstract

**Hintergrund:**

Für den Fachbereich Dermatologie steht gerade in den letzten Jahren ein zunehmendes Spektrum an Systemtherapien zur Verfügung. Bei einigen dieser Medikamente handelt es sich um einen Off-label-Use, was beispielsweise zu Problemen bei der Kostenerstattung führen kann. Dieser Beitrag soll daher einen Überblick über die derzeit zugelassenen Systemtherapien in der Dermatologie bieten und weitere Alternativen wie Compassionate Use und Early-Access-Programme aufzeigen.

**Material und Methoden:**

Die Recherche der zugelassenen Medikamente in Deutschland wurde online in der Datenbank für Arzneimittel des Bundesinstituts für Arzneimittel und Medizinprodukte durchgeführt. Zudem erfolgte ein Abgleich mit den Angaben in der Roten Liste.

**Ergebnisse:**

Für insgesamt 50 dermatologisch relevante Krankheitsbilder werden tabellarisch die jeweils zugelassenen Systemtherapien dargestellt.

**Diskussion:**

Es kann festgestellt werden, dass die enormen Weiterentwicklungen der letzten Jahre und die zunehmend gute Evidenz in vielen Fällen trotz oftmals fehlender klinischer Studien im Fachbereich der Dermatologie sehr Erfolg versprechende systemische Behandlungskonzepte bieten. Jedoch kann der oft notwendige Off-label-Use Schwierigkeiten im klinischen Alltag verursachen. Der behandelnde Arzt sollte ebenso wie der Patient daher immer informiert sein, wenn es sich bei einer geplanten Therapie um einen Off-label-Use handelt. Es sollten zuvor zugelassene Alternativen in Erwägung gezogen werden, und eine adäquate Aufklärung der Patienten sollte erfolgen.

## Abstract (en)

**Background:**

In recent years, an increasing spectrum of systemic therapies has become available in the field of dermatology. Some of these drugs are used off-label, which for example can lead to problems with reimbursement. This article is therefore intended to provide an overview of the currently approved systemic therapies in dermatology and to point out further alternatives such as Compassionate Use and Early Access Programs.

**Materials and methods:**

The search for approved drugs in Germany was conducted online in the database for drugs of the Federal Institute for Drugs and Medical Devices. In addition, a comparison was made with the information provided from the *Rote Liste*.

**Results:**

For a total of 50 dermatologically relevant diseases, the respective approved system therapies are presented in tabular form.

**Conclusions:**

It can be stated that the enormous developments over the last few years and the increasingly good evidence offer in many cases very promising systemic treatment concepts despite the frequent lack of clinical studies in the field of dermatology. However, the often necessary off-label use can cause difficulties in everyday clinical practice. The attending physician should therefore always be informed if a planned therapy involves off-label use. Previously approved alternatives should be considered and patients should be adequately informed.

Der sog. Off-label-Use beschreibt den Einsatz eines zugelassenen Arzneimittels außerhalb der in der Zulassung genannten Indikationen (Abb. 1). Die oftmals fehlende Kostenübernahme zulasten der gesetzlichen Krankenkassen (GKV) stellt dabei sowohl Arzt als auch Patient im klinischen Alltag vor Schwierigkeiten [3].

In dem Fachbereich der Dermatologie gewinnen im letzten Jahrzehnt verschiedene Systemtherapien zunehmend an Bedeutung. Exemplarisch können Medikamente aus der Gruppe der Biologika oder spezifischen Immuntherapeutika genannt werden, die für verschiedene dermatologische Krankheitsbilder empfohlen und genutzt werden [11]. Hier bieten sich neue Möglichkeiten, Therapien individueller auszuwählen und anzupassen [11]. Das zunehmend breite Spektrum an Systemtherapien findet sich auch als Empfehlungen in Lehrbüchern und Leitlinien wieder. Oft fehlt aber die Information, ob es sich um eine hierfür zugelassene Therapie oder einen Off-label-Use handelt. Der Gemeinsame Bundesausschuss (G-BA) beschreibt: „Unter Off-Label-Use wird der zulassungsüberschreitende Einsatz eines Arzneimittels außerhalb der von den nationalen oder europäischen Zulassungsbehörden genehmigten Anwendungsgebiete (Indikationen, Patientengruppen) verstanden.“ Grundsätzlich obliegt die Entscheidung über die Therapie einer Erkrankung dem behandelnden Arzt. Er kann bei entsprechender Begründung die Medikation wählen, unabhängig von dem jeweiligen Zulassungsstatus. Der Off-label-Use ist in vielen Fachbereichen der Medizin unausweichlich, da bei einigen Erkrankungen, insbesondere bei sog. Orphan Diseases, zugelassene Therapien fehlen oder im Einzelfall kein ausreichendes Therapieansprechen zeigen [2]. Orphan Diseases sind seltene Erkrankungen mit einer Prävalenz von weniger als 5 von 10.000 Einwohnern [7]. Da in Deutschland die Erstattung von Medikamenten durch die GKV an die Verordnungspflicht und an das Label gekoppelt ist, werden für seltene Erkrankungen Therapien häufig nicht erstattet, obwohl im Einzelfall der Off-label-Use sogar eine ärztliche Verpflichtung darstellen kann. Dies bedeutet beispielsweise, dass bei einer lebensbedrohlichen Erkrankung oder bei lebenseinschränkenden Folgen bei ausbleibender Behandlung das einzig wirksame Medikament eingesetzt werden muss, selbst wenn es sich hier um einen Off-label-Use handelt [10] (s. Gerichtsurteil vom 30.05.1990 LG Köln [12]). Problematisch ist in diesem Kontext oft die Kostenerstattung zulasten der GKV, da diese grundsätzlich an die Zulassung geknüpft ist. Diese orientiert sich an dem § 2 Abs. 1 Satz 3, § 70 Abs. 1 Satz 1 und § 72 Abs. 2, SGB (Sozialgesetzbuch) V sowie insbesondere an dem § 12, Abs. 1, SGB V (Wirtschaftlichkeit) [1]. Nur in ausgewiesenen Einzelfällen sind Verordnungen im Off-label-Use zulasten der GKV verordnungsfähig. Dies ist nur dann zulässig, wenn

- es um die Behandlung einer schwerwiegenden, lebensbedrohlichen oder die Lebensqualität auf Dauer nachhaltig beeinträchtigenden Erkrankung geht *und*
- keine andere zugelassene Therapie verfügbar ist *und*
- aufgrund der Datenlage die begründete Aussicht besteht, dass mit dem betreffenden Präparat ein Behandlungserfolg (kurativ oder palliativ) erzielt werden kann.

Damit Letzteres angenommen werden kann, müssen Forschungsergebnisse vorliegen, die erwarten lassen, dass die Arzneimittel für die betreffende Indikation zugelassen werden können. Davon kann ausgegangen werden, wenn

- entweder die Erweiterung der Zulassung bereits beantragt ist und die Ergebnisse einer kontrollierten klinischen Prüfung der Phase III (gegenüber Standard oder Placebo) veröffentlicht sind und eine klinisch relevante Wirksamkeit respektive einen klinisch relevanten Nutzen bei vertretbaren Risiken belegen *oder*
- außerhalb eines Zulassungsverfahrens gewonnene Erkenntnisse veröffentlicht sind, die über Qualität und Wirksamkeit des Arzneimittels in dem neuen Anwendungsgebiet zuverlässige, wissenschaftlich nachprüfbare Aussagen zulassen und aufgrund derer in den einschlägigen Fachkreisen Konsens über einen voraussichtlichen Nutzen in dem vorgenannten Sinne besteht.

Werden diese Kriterien nicht erfüllt, droht grundsätzlich die Gefahr, dass der verordnende Arzt in Regress genommen wird. Auch der Patient kann Ansprüche gegen den behandelnden Arzt geltend machen, wenn die Krankenkasse ein Medikament im Off-label-Use nicht erstattet. Dies kann geltend gemacht werden, auch wenn das entsprechende Medikament kostengünstiger ist oder sogar weniger Nebenwirkungen hat im Vergleich zu den hierfür zugelassenen Medikamenten [15].

Dieser Beitrag soll daher einen Überblick über die derzeit zugelassenen Systemtherapien relevanter Erkrankungen in der Dermatologie bieten und die Problematik des Off-label-Use sowie weitere Alternativen aufzeigen.

## Material und Methoden

Die Recherche der in Deutschland zugelassenen Medikamente für dermatologische Krankheitsbilder wurde online in dem Arzneimittel-Informationssystem (AMIS) mit letzter Aktualisierung des April 2020 durchgeführt. Hierbei handelt es sich um die Datenbank für Arzneimittel des Bundesinstituts für Arzneimittel und Medizinprodukte (BfArM). Es wurden hierfür relevante dermatologische Erkrankungen überprüft ohne Anspruch auf Vollständigkeit. Für jede dieser einzelnen Erkrankungen wurden alle zugelassenen Therapien herausgefiltert und nach Systemtherapie und topischer Therapie sortiert.

Um Unvollständigkeit zu vermeiden, erfolgte zudem ein Abgleich mit den Einträgen in der Roten Liste von 2020. Diese Vorgehensweise wurde auf Richtigkeit von 3 Ärzten unabhängig voneinander überprüft.

In dieser Arbeit wurde der Fokus ausschließlich auf Systemtherapien gelegt, weshalb die topischen Therapien keine Erwähnung finden. Infektionskrankheiten wurden ebenfalls nicht mit aufgenommen, da hier Systemtherapien wie Antibiotika und Antimykotika zum Einsatz kommen, die in der Regel nicht krankheitsspezifisch, sondern erregerspezifisch sind. Im klinischen Alltag erfolgt die Auswahl der eingesetzten Medikamente meist individuell je nach Erreger und Resistogramm. Als einzige Ausnahme haben wir Doxycyclin bei Rosazea aufgeführt, da es hier ein Medikament mit 40 mg retardierten Hartkapseln gibt, das ausschließlich für die Behandlung der Rosazea zugelassen wurde.

Nach erfolgter Recherche wurden die Wirkstoffnamen der Systemtherapien aufgelistet. Einige Medikamente, wie beispielsweise Antihistaminika, wurden in Gruppen eingeteilt und für die bessere Lesbarkeit in der Tabelle entsprechend zusammengefasst.

## Ergebnisse

Wir haben für insgesamt 50 dermatologisch relevante Krankheitsbilder zugelassene Systemtherapien zusammengefasst. Die Tab. 1 zeigt die Ergebnisse, aufgelistet in alphabetischer Reihenfolge. Wirkstoffgruppen wie systemische Glukokortikoide oder Antihistaminika wurden gesammelt aufgelistet (Tab. 1).

**Table 1:** 

Krankheitsbild	Zugelassene Systemtherapeutika
Akne	Chlormadinon/Ethinylestradiol
	Cyproteronacetat/Ethinylestradiol
	Dienogest/Ethinylestradiol
	Cyproteronacetat
	Isotretinoin
Acne inversa (Hidradenitis suppurativa)	Adalimumab
Angioneurotisches (Quincke) Ödem	Methylprednisolon/Prednisolon/Prednison
Atopische Dermatitis	Ciclosporin
	Dupilumab
	Prednisolon/Prednison
Basalzellkarzinom	Sonidegib
	Vismodegib
Bullöses Pemphigoid	Azathioprin
	Dapson
	Prednisolon/Prednison
Cryopyrin-assoziierte periodische Syndrome	Anakinra
	Canakinumab
Dermatitis herpetiformis Duhring	Dapson
Dermatofibrosarcoma protuberans	Imatinib
Dermatomyositis	Azathioprin
	Dexamethason/Methylprednisolon/Prednisolon/Prednison
Dyskeratosis follicularis (Morbus Darier)	Acitretin
Epidermolysis bullosa	Methylprednisolon/Prednisolon/Prednison
Erythema elevatum et diutinum	Dapson
Erythema exsudativum multiforme	Methylprednisolon/Prednisolon/Prednison
Erythema nodosum	Methylprednisolon/Prednisolon/Prednison
Granuloma anulare	Dapson
Morbus Fabry	Agalsidase alfa und beta
	Migalastat
Hämangiom	Prednisolon/Prednison
	Propranolol
Handekzem	Alitretinoin
Hyperhidrose	Bornaprin
	Methantheliniumbromid
Ichthyosis	Acitretin
Kaposi-Sarkom	Dacarbazin
	Doxorubicin
	Interferon alfa-2a
	Paclitaxel
Leishmaniose	Amphotericin B
	Pentamidin
Lichen ruber	Acitretin
	Prednisolon/Prednison
Lupus erythematodes	Azathioprin
	Belimumab
	Dexamethason/Methylprednisolon/Prednisolon/Prednison
	Chloroquin
	Cyclophosphamid
	Hydroxychloroquin
Malignes Melanom	Binimetinib
	Carmustin
	Cobimetinib
	Dabrafenib
	Dacarbazin
	Encorafenib
	Interferon alfa-2a und -2b
	Ipilimumab
	Lomustin
	Melphalan
	Nivolumab
	Pembrolizumab
	Talimogen laherparepvec
	Trametinib
	Vemurafenib
Mastozytose	Midostaurin
Merkel-Zell-Karzinom	Avelumab
Morbus Adamantiades-Behçet	Apremilast
	Azathioprin
	Ciclosporin
	Prednisolon/Prednison
Mycosis fungoides	Bexaroten
	Methoxsalen
	Mogamulizumab
Parapsoriasis	Prednisolon
	Prednison
Pemphigus (vulgaris)	Azathioprin
	Dapson
	Dexamethason/Methylprednisolon/Prednisolon/Prednison
	Rituximab
Pityriasis rubra pilaris	Acitretin
	Prednisolon/Prednison
Plattenepithelkarzinom	Bleomycin
	Carboplatin
	Cemiplimab
	Cetuximab
	Cisplatin
	Docetaxel
	Fluorouracil
	Temoporfin
	Vindesinsulfat
Polyarteriitis nodosa	Azathioprin
	Dexamethason/Methylprednisolon/Prednisolon/Prednison
Prurigo pigmentosa	Dapson
Psoriasisarthritis	Abatacept
	Apremilast
	Certolizumab
	Cyclophosphamid
	Etanercept
	Golimumab
	Infliximab
	Ixekizumab
	Leflunomid
	Methotrexat
	Secukinumab
	Tofacitinib
	Ustekinumab
Psoriasis pustulosa	Acitretin
	Dapson
	Methoxsalen
	Prednisolon/Prednison
Psoriasis vulgaris	Acitretin
	Adalimumab
	Apremilast
	Brodalumab
	Certolizumab
	Ciclosporin
	Dimethylfumarat
	Etanercept
	Guselkumab
	Infliximab
	Ixekizumab
	Methotrexat
	Prednisolon/Prednison/Triamcinolon
	Risankizumab
	Secukinumab
	Tildrakizumab
	Ustekinumab
Pyoderma gangraenosum	Prednisolon/Prednison
Raynaud-Syndrom	Nifedipin
	Iloprost
Rosazea	Doxycyclin
Sarkoidose	Prednisolon/Prednison/Methylprednisolon
Sézary-Syndrom	Mogamulizumab
	Prednisolon/Prednison
Skabies	Ivermectin
Sklerodermie	Bosentan
	Methylprednisolon
	Penicillamin
	Prednisolon
	Prednison
Stevens-Johnson-Syndrom/toxisch epidermale Nekrolyse	Methylprednisolon/Prednisolon/Prednison
Urtikaria	Antihistaminika
	Methylprednisolon/Prednisolon/Prednison
Vaskulitis	Azathioprin
	Cyclophosphamid
	Dapson
	Dexamethason/Methylprednisolon/Prednisolon/Prednison
	Rituximab
Vitiligo	Methoxsalen

Nicht für jede dermatologische Erkrankung gibt es zugelassene Systemtherapien. Als relevante Beispiele können unter anderem Kalziphylaxie, CREST-Syndrom, Livedovaskulopathie oder Neurofibromatose genannt werden. Erkrankungen ohne zugelassene Systemtherapie wurden in der Tabelle nicht explizit aufgeführt.

## Diskussion

Die enormen Weiterentwicklungen der Systemtherapeutika in dem Fachbereich Dermatologie in den letzten Jahren bieten viele Chancen für sehr Erfolg versprechende, individuelle Behandlungskonzepte. Bei der Therapieplanung sollte aber unter anderem ein Off-label-Use berücksichtigt werden. Es ist anzunehmen, dass häufig ein Off-label-Use erfolgt, ohne dass es dem behandelnden Arzt bewusst ist, dass es sich überhaupt um einen solchen handelt. Leider werden nicht in allen aktuellen Lehrbüchern und Leitlinien die Empfehlungen systemischer Therapien als Off-label-Use gekennzeichnet. Als ein Beispiel hierfür kann S2k-Leitlinie Mycosis fungoides genannt werden. Abhängig von dem Stadium werden tabellarisch die empfohlenen Therapien aufgelistet. Zugelassen sind in Deutschland lediglich Bexaroten, Methoxsalen und Mogamulizumab. Als Off-label-Use werden zwar Doxorubicin, Brentuximab und Pralatrexat gekennzeichnet; diese Deklaration fehlt dann aber bei Acitretin, Methotrexat, Gemcitabin, Alemtuzumab, Fludarabin, Cladribin und Cyclophosphamid [6]. Ein Off-label-Use liegt auch vor, wenn sich Dosierungen von Medikamenten außerhalb des zugelassenen Bereichs für diese Erkrankung befinden. Beispielhaft ist hier die 4‑fach-Dosis eines Antihistaminikums bei der chronisch spontanen Urtikaria zu nennen. Dass es sich hierbei um einen Off-label-Use handelt, ist weder in der deutschen noch in der europäischen Leitlinie entsprechend gekennzeichnet. Darüber hinaus beträgt die zugelassene Dosierung von Omalizumab 300 mg s.c. alle 4 Wochen. Werden die Dosis und/oder das Intervall bei fehlendem Ansprechen verändert, dann handelt es sich um einen Off-label-Use. In der deutschen und europäischen Leitlinie zur Urtikaria ist die Dosierung von Omalizumab nicht explizit benannt [21, 22], jedoch wird in der deutschsprachigen Übersetzung der europäischen Leitlinie von 2018 eine Dosierung von 150–300 mg empfohlen [23]. Ein Beispiel für eine Leitlinie, in der ein Off-label-Use stringent benannt wird, ist die S2k-Leitline für blasenbildende Autoimmunerkrankungen [16]. Für Patienten mit bullösem Pemphigoid sind in Deutschland Azathioprin, Prednisolon und Prednison als Systemtherapien zugelassen. In der Leitlinie werden andere empfohlene Therapieoptionen wie Dapson, Doxycyclin, Methotrexat, Mycophenolat-Mofetil, Cyclophosphamid und intravenöse Immunglobuline eindeutig als Off-label-Use gekennzeichnet [16]. Erwähnenswert in Bezug auf das Doxycyclin ist eine Neuerung, die am 10.12.2020 in Kraft getreten ist. Der Gemeinsame Bundesausschuss (G-BA) hat Doxycyclin zur Behandlung des bullösen Pemphigoids als erlaubten Off-label-Use zugelassen. Der Einsatz von Doxycyclin ist bei älteren Patienten dann zu empfehlen, wenn unter Azathioprin oder systemischen Glukokortikoiden eine Verschlechterung des Allgemeinzustandes zu erwarten ist. Hierfür sind allerdings nur Doxycyclin-haltige Arzneimittel von Herstellern verordnungsfähig, die eine entsprechende Haftungserklärung abgegeben haben [8]. An diesem Beispiel wird deutlich, dass sich Leitlinien, die in der Regel eine Gültigkeit über 5 Jahre haben, nicht immer eignen einen aktuellen Überblick über den aktuellen Zulassungsstatus zu bieten. Um dies gewährleisten zu können, müssten beispielsweise Tabellen fortlaufend aktualisiert werden. Dies könnte zukünftig eine Aufgabe sein, die durch die Fachgesellschaft übernommen wird.

Die aktuell verfügbaren Daten zu der Häufigkeit eines Off-label-Use sind sehr limitiert. In der Onkologie beispielsweise beträgt der Einsatz von Medikamenten im Off-label-Use etwa 60 %, in der Kinderonkologie sind es sogar 90 % [17]. In einer französischen Studie der Universitätshautklinik in Rouen wurde die Häufigkeit des Off-label-Use von Februar bis April 2001 erfasst und lag bei 14 % [14]. Einschränkend muss beachtet werden, dass hier sowohl topische als auch systemische Therapien aufgenommen wurden. In einer Studie aus Freiburg lag der Off-label-Use von Systemtherapien in der Dermatologie bei 15 % [3]. In anderen Studien liegt der Einsatz von Systemtherapien im Off-label-Use je nach Indikationsgebiet zwischen 30 und 90 % [2]. In einer retrospektiven Auswertung über Off-label-Use in 2 dermatologischen deutschen Hochschulambulanzen von 2010 bis 2012 zeigte sich, dass von den Kostenübernahmeanträgen 56,8 % bewilligt wurden, nach Widersprüchen gegen die Ablehnung einer Kostenübernahme sogar 75 %. Einschränkend muss erwähnt werden, dass nur dermatologische Spezialsprechstunden wie die Autoimmun- und Urtikariasprechstunden ausgewertet wurden [18].

Vor einem Off-label-Use sollte immer eine ausführliche Aufklärung der Patienten erfolgen. Hierbei muss u. a. über den zulassungsrechtlichen Status und die möglicherweise bislang unbekannten Risiken aufgeklärt werden. Problematisch in diesem Kontext ist, dass es insbesondere bei seltenen Erkrankungen häufig keine Studien gibt, die zu einer solchen Zulassung führen könnten. Der Weg bis zu einer Zulassung eines Medikaments für ein Krankheitsbild ist sehr aufwendig. Zunächst laufen präklinische Studien, die an Zellkulturen und/oder Tieren durchgeführt werden, um die Sicherheit einer Substanz zu prüfen. Zeigt sich eine Substanz entsprechend sicher, werden klinische Studien und somit Testungen am Menschen geplant. Eine Ethikkommission muss vor jeder Studie ihre Zustimmung geben [19]. In Phase-I-Studien wird dann erstmals ein Medikament in geringen Dosen einer kleineren Gruppe freiwilliger, gesunder Probanden gegeben. Es wird dabei überprüft, ob sich das Medikament bei Menschen genauso verhält wie zuvor in präklinischen Studien. In Phase-II- und -III-Studien werden dann Wirksamkeit, optimale Dosierung und Verträglichkeit des Präparates bei Patienten getestet. Dabei werden die Studienteilnehmer in Gruppen eingeteilt, wobei ein Teil das neue Medikament bekommt und der andere Teil Standardmedikamente oder Placebo. Diese Studientypen werden, falls möglich, doppelt verblindet durchgeführt. Anschließend muss der Hersteller einen kostenpflichtigen Antrag bei der Zulassungsbehörde stellen. Hierzu reicht er Unterlagen über die technische Qualität des Arzneimittels und sämtliche vorklinische und klinische Studienergebnisse ein. In Europa ist hierfür die europäische Arzneimittelagentur European Medicines Agency (EMA) in Amsterdam zuständig. Jedoch kann der Antrag auch bei einer nationalen Zulassungsbehörde gestellt werden. In Deutschland sind das Bundesinstitut für Arzneimittel und Medizinprodukte (BfArM) in Bonn und das Paul-Ehrlich-Institut (PEI) in Langen bei Frankfurt am Main zuständig; in den USA entscheidet die Food and Drug Administration (FDA) als zentrale Behörde über die Zulassung von Medikamenten. Nach der Zulassung erfolgen dann noch Phase-IV-Studien, die auch als Sicherheitsstudien bezeichnet werden. Diese können freiwillig sein oder von der Zulassungsbehörde vorgeschrieben werden. In Phase-IV-Studien werden meist große Patientengruppen und längere Zeiträume beobachtet. Insbesondere eher seltene Nebenwirkungen können so detektiert werden [19]. Es wird deutlich, dass es sich bis zur Zulassung eines Medikamentes um eine sehr langwierige und kostenintensive Prozedur handelt, weshalb pharmakologische Unternehmen bevorzugt auf Zulassungen bei großen Absatzmärkten und Indikationen abzielen.

Es gibt aber auch Möglichkeiten, außerhalb von klinischen Studien Patienten den Zugang zu potenziell lebensrettenden Medikamenten zu ermöglichen. Unter der Bezeichnung Compassionate Use (wörtlich „Anwendung aus Mitgefühl“) Program (CUP) versteht man eine Härtefallregelung, bei der (noch) nicht zugelassene Medikamente an Patientengruppen vergeben werden können, die an Erkrankungen leiden, die zu einer schweren Behinderung führen würden oder als lebensbedrohend gelten und die mit einem zugelassenen oder genehmigten Arzneimittel nicht zufriedenstellend behandelt werden können [1]. In Deutschland meldet der Hersteller beim BfArM oder PEI ein Medikament für solch ein CUP an. Hier wird genau definiert, welche Diagnose für die Behandlung vorliegen muss. Trifft dies zu, kann das Medikament kostenfrei von dem behandelnden Arzt bei dem Pharmahersteller angefordert werden.

Bei dem Early Access Program (EAP) kann ein Hersteller eines vielversprechenden Medikaments Patienten den frühzeitigen Zugang zu seinem noch nicht zugelassenen Medikament ermöglichen. Dies betrifft z. B. Patienten, die das Medikament in Rahmen einer klinischen Studie eingenommen haben und die Einnahme gerne fortführen würden. Diese Programme werden oftmals genauso genehmigt wie die klinischen Studien selbst; die Kosten liegen bei den Herstellern. Die Vorteile, die sich dem jeweiligen pharmakologischen Unternehmen bieten, liegen u. a. in der Nutzung der daraus gewonnenen Patientenerfahrungen [13]. Die Genehmigung der EAPs unterliegt nationalen Vorschriften, die sich an den Richtlinien der EMA orientieren. In Deutschland finden sich die nationalen Richtlinien in der Arzneimittel-Härtefall-Verordnung wieder.

Nicht verschreibungspflichtige Medikamente können in Ausnahmefällen zulasten der GKV erstattet werden. Hierfür gibt es eine G‑BA-Ausnahmeliste nach § 34 Abs. 1 Satz 2 SGB V (OTC-Übersicht), Anlage I, Abschnitt F der Arzneimittel-Richtlinie, Stand (letzte Änderung in Kraft getreten am 09.11.2018 [9]). Beispielsweise werden Antihistaminika im Falle einer schweren, rezidivierenden Urtikaria von der GKV erstattet. Im stationären Bereich gelten zwar grundsätzlich die gleichen Bedingungen wie im ambulanten Bereich, aber durch die pauschalisierten Entgeltsysteme nach § 7 Abs. 1 Satz 1 Krankenhausentgeltgesetz (KHEntgG) werden stationäre Aufenthalte durch Fallpauschalen und Zusatzentgelte abgerechnet und vergütet. Vergütet werden alle Maßnahmen, die im Einzelfall nach Art und Schwere der Krankheit für die medizinisch zweckmäßige und ausreichende Versorgung des Patienten notwendig sind [5].

Eine weitere Option für die Praxis kann am Beispiel Adalimumab aufgezeigt werden. Hierbei handelt es sich um ein Medikament, das für die Behandlung des Morbus Adamantiades-Behçet nicht zugelassen ist. Allerdings besteht eine Zulassung für die Behandlung der nichtinfektiösen Formen der Uveitis bei erwachsenen Patienten, die nur unzureichend auf Glukokortikoide angesprochen haben. Da die Uveitis ein kardinales Symptom des Morbus Adamantiades-Behçet darstellt, kann somit oft ein Off-label-Use vermieden werden [20].

Im klinischen Alltag werden regelmäßig Medikamente im Off-label-Use verschrieben, oft ohne zuvor einen Antrag auf Übernahme der Kosten bei der GKV gestellt zu haben. Hier droht grundsätzlich die Gefahr von Regressen. Auf den ersten Blick widersprüchlich erscheint die Verpflichtung, Systemtherapien aus dem Off-label-Bereich anzuwenden, wenn es das einzige wirkungsvolle Medikament ist, das zur Verfügung steht, um eine schwerwiegende, lebensbedrohliche oder die Lebensqualität auf Dauer nachhaltig beeinträchtigende Erkrankung zu behandeln. So kam es 1990 in Köln zu einem Gerichtsverfahren, bei dem es um die Frage ging, ob bei einem 1,5-jährigen Kind mit Herpes-Virus-Enzephalitis Aciclovir früher hätte eingesetzt werden müssen, obwohl dies zu diesem Zeitpunkt zur Behandlung noch nicht zugelassen war. Das Gericht kam zu dem Urteil, dass das Medikament bereits bei dem Verdacht auf eine Herpes-Enzephalitis Aciclovir hätte eingesetzt werden müssen, da es die einzig Erfolg versprechende Therapie darstellte, deren Wirksamkeit zu diesem Zeitpunkt bereits wissenschaftlich belegt war. Eine fehlende Zulassung darf also kein Hinderungsgrund zum Einsatz des Medikamentes sein, insbesondere wenn es sich um eine schwerwiegende, lebensbedrohliche Krankheit handelt und es keine alternative Therapie gibt [4].

Zusammenfassend kann feststellt werden, dass Ärzte einerseits verpflichtet sein können, auch Medikamente, die nicht zugelassen sind, zu verordnen, wenn diese lebensrettend sind oder die Lebensqualität erheblich verbessern, wenn keine anderen Medikamente zu Verfügung stehen oder aufgrund von Kontraindikationen nicht eingesetzt werden können. Andererseits droht ein Regress, wenn die oben genannten Kriterien nicht erfüllt sind. Obwohl diese Aspekte sehr wichtig sind, gibt es aktuell keine verbindliche Regelung für eine einheitliche und konsequente Kennzeichnung eines Off-label-Use in Leitlinien und Lehrbüchern.

**Figure 1:**
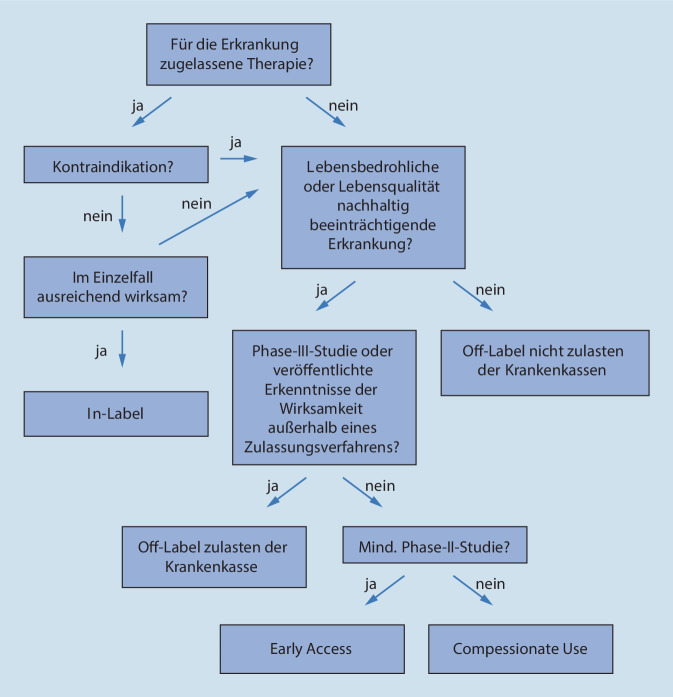

## Fazit für die Praxis

- Für eine große Anzahl dermatologischer Krankheitsbilder ist unter der aktuellen Off-label-Use-Regelung eine Verordnung zulasten der GKV (gesetzliche Krankenkassen) nicht möglich.
- Ärzten und Patienten sollte es bewusst sein, wenn Medikamente im Off-label-Use eingesetzt werden.
- In Leitlinien und Lehrbüchern sollte bei den Therapieempfehlungen der Off-label-Use eindeutig gekennzeichnet sein.
- Ärzte sollten über aktuelle Entwicklungen zu Compassionate Use und Early-Access-Programmen regelmäßig informiert werden.
